# Exploring evolution of maximum growth rates in plankton

**DOI:** 10.1093/plankt/fbaa038

**Published:** 2020-09-04

**Authors:** Kevin J Flynn, David O F Skibinski

**Affiliations:** Plymouth Marine Laboratory, Prospect Place, West Hoe, Plymouth PL1 3DH, UK; Swansea University Medical School, Swansea University, Swansea, SA2 8PP, UK

**Keywords:** NPZ model, plankton evolution, extinction, climate change

## Abstract

Evolution has direct and indirect consequences on species–species interactions and the environment. However, Earth systems models describing planktonic activity invariably fail to explicitly consider organism evolution. Here we simulate the evolution of the single most important physiological characteristic of any organism as described in models—its maximum growth rate (μ_m_). Using a low-computational-cost approach, we incorporate the evolution of μ_m_ for each of the plankton components in a simple Nutrient-Phytoplankton-Zooplankton -style model such that the fitness advantages and disadvantages in possessing a high μ_m_ evolve to become balanced. The model allows an exploration of parameter ranges leading to stresses, which drive the evolution of μ_m._ In applications of the method we show that simulations of climate change give very different projections when the evolution of μ_m_ is considered. Thus, production may decline as evolution reshapes growth and trophic dynamics. Additionally, predictions of extinction of species may be overstated in simulations lacking evolution as the ability to evolve under changing environmental conditions supports evolutionary rescue. The model explains why organisms evolved for mature ecosystems (e.g. temperate summer, reliant on local nutrient recycling or mixotrophy), express lower maximum growth rates than do organisms evolved for immature ecosystems (e.g. temperate spring, high resource availability).

## INTRODUCTION

There is a need in ecological models to clarify the role of evolution of those traits that have the most profound effects on simulation output at a general level (Bell, 2013; Merila and Hendry, 2014). This is especially so with respect to plankton given their high growth rates and their major role in global primary production (Field *et al*., 1998). While the evolution of organisms has been considered in plankton research using models (Jiang *et al*., 2005; Follows *et al*., 2007; Sauterey *et al*., 2015), very little effort has been made to simulate the process and consequences of evolution itself on organisms and functional types, and thence on trophic interactions.

Major challenges lie in identifying key traits to evolve, and then upon what basis to model their evolution, complicated as the topic is by the multi-faceted physiological interactions associated with any given trait. While most emphasis in modelling has been hitherto expended on bottom-up trait trade-offs (especially considering resource acquisition) such simple approaches fail to easily explain ecology (Raven *et al*., 2005; Sommer *et al*., 2017). It is also clear that the evolution of individual organisms or functional groups will affect and be affected by changes in the whole ecosystem and food web and will thus affect trophic dynamics. Modelling evolution of just one trophic level or of one functional group in such a system is thus unlikely to advance understanding; we need an approach to apply across an ecosystem model. There is then the challenge of simulating evolution itself. Is this to be considered using an individual-based modelling (IBM) approach, or biomass-based, and how many traits can be simulated without overwhelming the computation?

For pragmatic reasons (computational, and also because the vast bulk of plankton models are not IBMs; Arora *et al*., 2013) we elected to derive a biomass-based approach. We also sought a single trait that operates across all organism groups, that is described in most if not all models, and that captures a critical aspect of organism function. We focus on the maximum potential growth rate (μ_m_). Arguably this is the most important feature contributing to competition success in dynamic settings, and a trait to which organisms show high sensitivity in models across all trophic levels. Despite its biological importance, the evolution of μ_m_ as a trait has not figured prominently in plankton modelling work.

The general perception is that there are fitness benefits to possessing a high growth rate potential under prevailing abiotic and biotic conditions (Droop, 1974; Reznick *et al*., 2002; Flynn, 2009). Related phytoplankton species show variation in μ_m_ indicating underlying genetic variation and evolution of this characteristic under different environmental conditions (Raven *et al*., 2005; Litchman *et* al., 2012; Edwards *et al*., 2013; Jin and Agusti, 2018). In experimental evolution studies on microorganisms, μ_m_ can be modified by selection resulting in genetic adaptation (Lenski *et al*., 1998; Wick *et al*., 2002). Boyd *et al*. (2013) comment that the great variability of maximum growth rates across phytoplankton isolates from different locations complicates modelling work. Understanding why there is such variation would significantly benefit plankton science.

The maximum growth rate is, in reality, defined by interacting physiological and life cycle processes that are a function of the genetic material of the organism. As an emergent property of whole organism physiology, it is an evolvable trait. However, in models, μ_m_ is typically set as a constant (e.g. Geider *et al*., 1996), or controlled by a constant that limits key processes, such as the maximum feeding rate (e.g. Gentleman *et al*., 2003). All else being equal, a high μ_m_ is the inevitably of competitive advantage. In models, there is no upper limit to the value that could be assigned to this parameter. However, in reality, the upward evolution of growth rate is restricted by a variety of physiological factors (Arendt, 1997; Molenaar *et al*., 2009; Dmitriew, 2011; Bosdriesz *et al*., 2015; Flynn and Mitra, 2016), and at the upper limit the advantage of high μ_m_ will be counterbalanced by associated costs. These costs may relate to metabolic trade-offs related to the synthesis of structural components (Arendt, 1997; Molenaar *et al*., 2009), optimizing protein synthesis and the balance of resources allocated to ribosomal and non-ribosomal proteins (Bosdriesz *et al*., 2015). Costs associated with high growth rate may also lead to increased mortality for a variety of reasons. During growth, fewer resources might be allocated to energy reserves, which may in turn lead to vulnerability to stress and mortality (Dmitriew, 2011). Rapid growth may also lead to cellular damage and mortality as a result of free radical generation and which might be proportionally greater at higher growth as result of progressive damage to mitochondrial membranes (Mangel and Munch, 2005; Monaghan *et al*., 2009).

We thus identified μ_m_ at the core of what could be viewed as a universal trait trade-off mechanism controlling growth potential. We present here a growth rate evolution model (GREM) to simulate the evolution of μ_m_, in which natural selection maximizes net growth for each member of a food web. This mechanism balances the advantage of possessing a high μ_m_ against the metabolic cost incurred to maintain this trait. To achieve this, in our models we re-designated μ_m_ for each organism (a value held as constant in traditional models) as a state variable, so the value could evolve over time. We demonstrate the behaviour and some implications of such simulations using a biomass-based NPZ-type of construct, considering evolution in response to generic stress. Climate change stressors also influence evolution (Chevin *et al*., 2010; Hoffmann and Sgro, 2011; Merila and Hendry, 2014) but plankton models used in climate change simulations for predictions of the impact of different climate change scenarios on planetary resources do not describe organism evolution (Flynn *et al*., 2015). We thus also consider some aspects pertaining to climate change, which is a feature of ecology at all scales and is frequently characterized by multi-stressors (Cahill *et al*., 2013), affecting organism growth and thence trophic interactions and biogeochemistry.

## METHOD

**Fig. 1:**
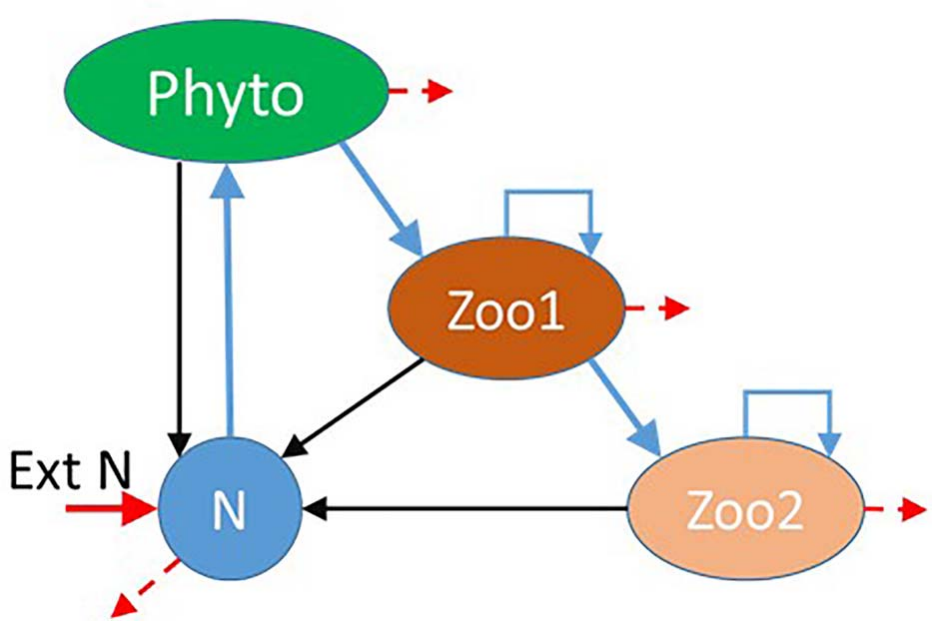
Schematic of NPZZ food-web. Light and dissolved inorganic nutrient (N) supports the primary producer Phyto, and thence consumers Zoo1 and Zoo2. Wastes and corpses are recycled (black arrows). Consumers can cannibalize, as indicated. A low rate of mixing in and out (red arrows, akin to a chemostat) introduces dissolved inorganic nitrogen (N, from ExtN) and removes some residual DIN and biomass. Full details are given in the Supplementary Appendix.

**Fig. 2:**
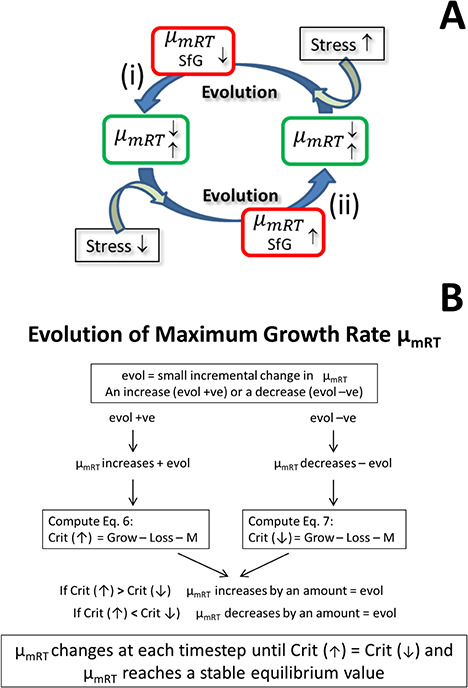
GREM concept. Panel A: the population average maximum growth rate at reference temperature (μ_mRT_) evolves by decreasing (i) or increasing (ii) to attain a new equilibrium value (green boxes) as both maximum growth rate at the reference temperature (μ_mRT_) and the SfG are optimized (red boxes) so minimizing stress. Panel B: the flow-schematic summarizing how evolution affects increases or decreases in μ_mRT_ by an amount evol in the GREM model. See text and Supplementary Appendix for further explanation.

**Fig. 3:**
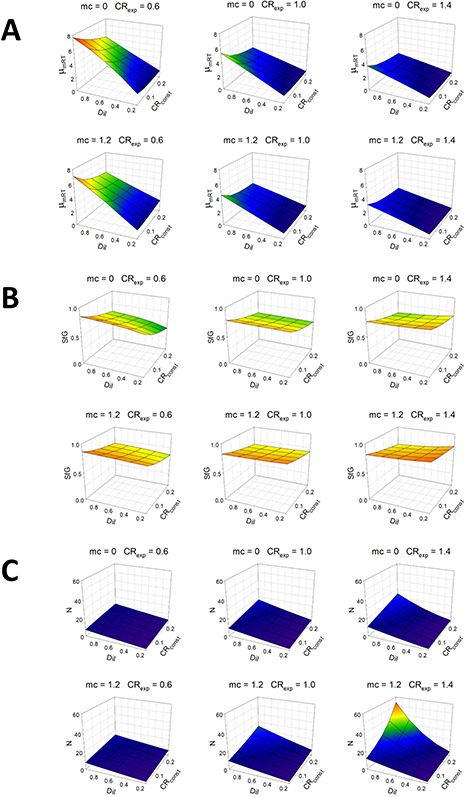
Mesh plots for Phyto growing in a N-limited chemostat where the realized growth rate equals the dilution rate (Dil). Outputs from 150 simulations were run to equilibrium over 2000 days in a factorial design with varying dilution rate (Dil; d^−1^), catabolic respiration constants (CR_const_ and CR_exp_; dimensionless) and mortality coefficient (mc; dimensionless); see Table I and Supplementary Table for parameter values. Shown are mesh surface plots for maximum growth rate at the reference temperature (μ_mRT_, panel A; d^−1^), SfG (panel B; dimensionless quotient) and residual nutrient (N, panel C; mgN m^−3^).

**Fig. 4:**
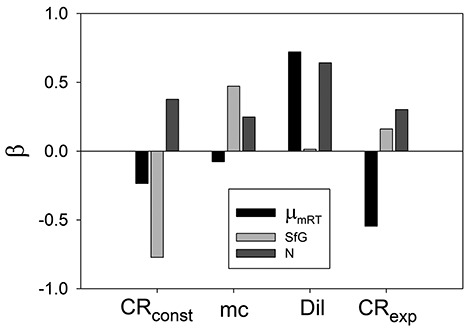
Regression statistics for Phyto chemostat. Data are from the simulations shown in Fig. 3. Shown are values of standardized regression coefficients (β values) for linear regression of the output variables maximum growth rate at the reference temperature (μ_mRT_), SfG and residual nutrient (N) on the parameters along the X-axis and which also feature in the mesh plots of Fig. 3.

**Fig. 5:**
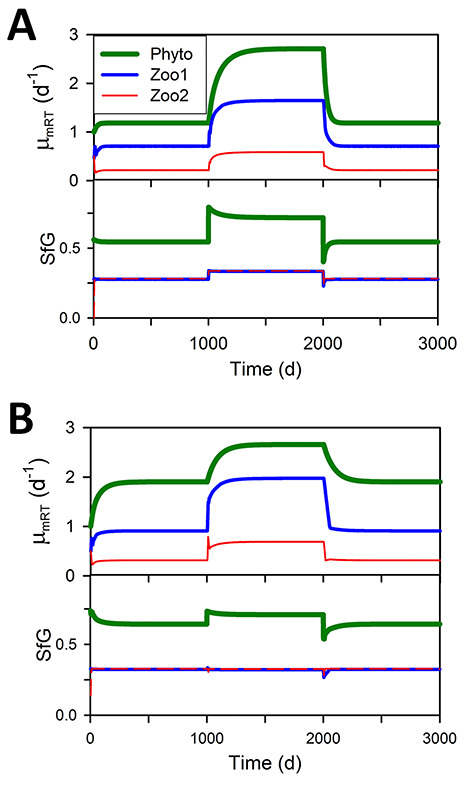
Evolution is reversible and maximum growth rate at the reference temperature (μ_mRT_) at equilibrium is lower for higher trophic levels. Panel A: time course of changes in μ_mRT_ and SfG of organisms within the NPZZ simulation subjected to a decrease in stress at time 1000, lowering catabolic respiration (CR_const_ changes from 0.25 to 0.1) followed by an increase in stress at time 2000 to the original level (CR_const_ changes from 0.1 to 0.25). Panel B: time course of changes in μ_mRT_ and SfG of organisms subjected to a decrease in stress at time 1000 due to changes in energy input from illumination via altering mixed layer water depth (Dep changes from 45 to 5) followed by an increase in stress at time 2000 to the original level (Dep changes from 5 to 45). In both A and B, SfG values for Zoo1 and Zoo2 are closely similar and overlap on the graphs.

**Fig. 6:**
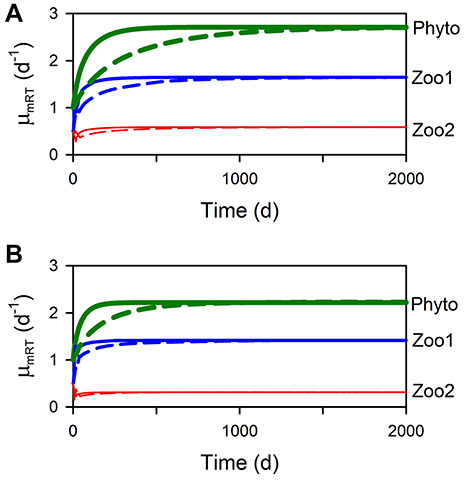
Evolutionary rate affects time to equilibrium but not equilibrium value. Two values of a constant λ determine the size of the evolutionary increment evol (Table I and Supplementary Appendix), and two values of the mortality coefficient (mc) determine the intensity of SfG dependent mortality. Panel A uses mc = 0 (lower mortality) and λ = 0.5 (higher evol) (solid line) or λ = 1 (lower evol) (dashed line). Panel B uses mc = 1 (higher mortality) and λ = 0.5 (solid line) or λ = 1 (dashed line).

**Fig. 7:**
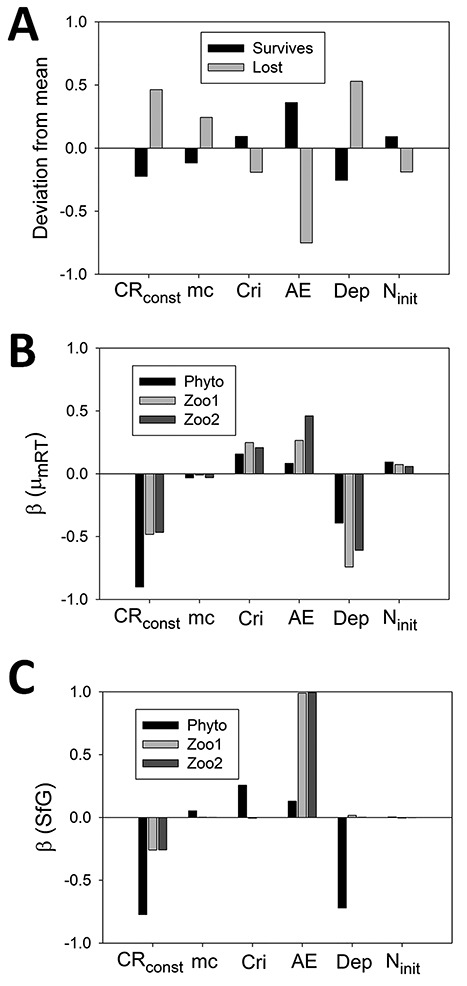
Regression statistics for NPZZ model. Detailed analysis of results from 200 simulations run to equilibrium over 2000 days (see also the summary in Fig. 8). The parameters shown along the X- axis are catabolic respiration constant (CR_const_; dimensionless), mortality coefficient (mc; d^−1^), prey capture rate (Cri; gN gN^−1^ d^−1^), AE(dimensionless), mixed layer water depth (Dep; m) and initial and external N (N_init_; mgN m^−3^). Values for the parameters were distributed evenly in parameter space over the simulations (see Supplementary Table for parameter value ranges). Panel A: Mean parameter values, expressed as deviations from the overall mean in standard deviations, are shown for simulations where Zoo2 is lost (N = 65) and survives (N = 135). For panels B and C, the standardized β values from regression analysis for the simulations where Zoo2 survives (N = 135) are analogous to those for the Phyto chemostat in Fig. 4.

**Fig. 8:**
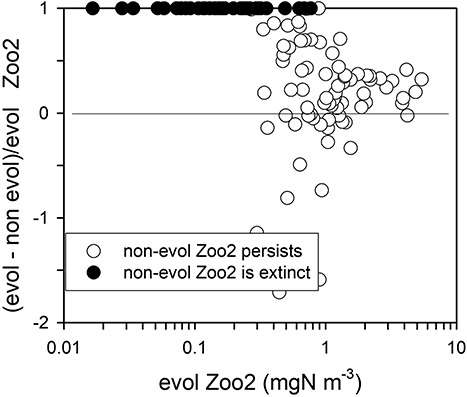
Summary of equilibrium values for Zoo2 in NPZZ simulations. Data come from the simulations run to produce Fig. 7, with parameter values distributed evenly over parameter space but run with evolution enabled (the value of evol is determined as in Supplementary Appendix), or with evolution disabled (evol is set to zero). X-axis shows the equilibrium biomass of Zoo2 in the evolving simulations. The Y-axis shows the difference between the Zoo2 biomass in evolving vs non-evolving simulations, normalized to the biomass in the evolving simulations (as given on the X-axis). Y-axis values of 1 thus indicate Zoo2 extinction in the non-evolving simulation; Y-axis = 0 indicates no difference in biomass between Zoo2 evolving and non-evolving; Y-axis < 0 indicates that non-evolving simulations had higher biomass than evolving simulations. Most extinctions occurred in low biomass non-evolving simulations of Zoo2.

**Fig. 9:**
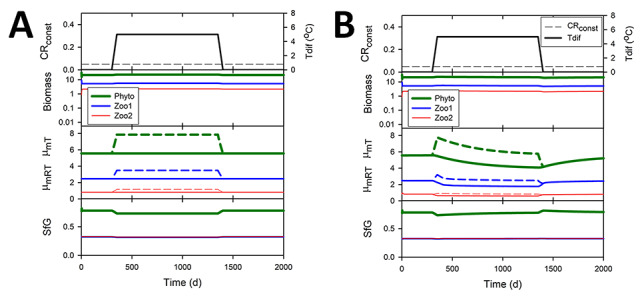
Growth within the NPZZ simulation with abrupt and persistent changes in temperature (T_dif_). The T_dif_ value increases from 0 to 5°C between 300 and 350 days, then decreases from 5 to 0°C between 1350 and 1400 days, while CR_const_ is held constant. Dashed lines, μ_mT_; solid lines, μ_mRT_. Panel A: Model output of a non-evolving system. Panel B: Model output of an evolving system.

**Fig. 10:**
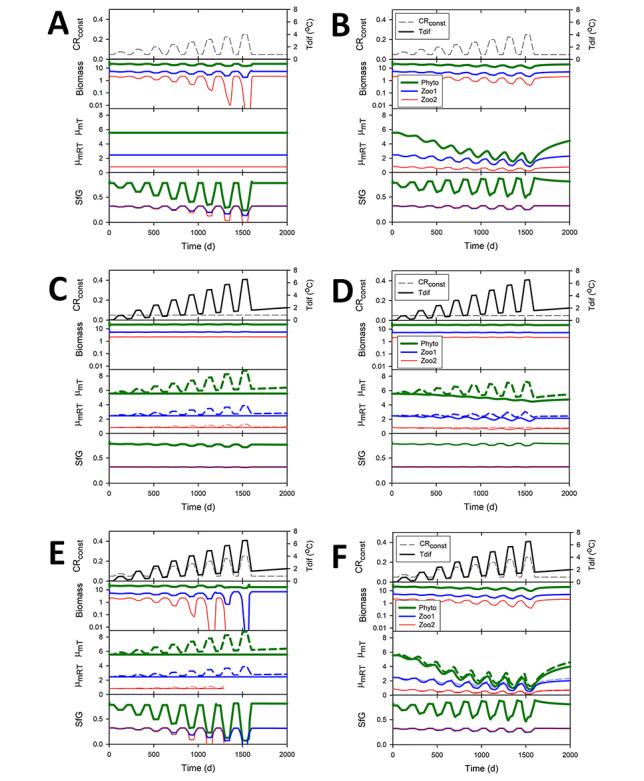
Growth under changing conditions. Changes over time in biomass, and physiological parameters for phytoplankton Phyto, Zoo1 and Zoo2 in the NPZZ simulation. The catabolic respiration (CR_const_) value is varied with increasing magnitude to represent changes in stress (panels A, B, E, F) or held constant (C, D). Temperature changes (T_dif_) are either held at zero (so μ_mRT_ and μ_mT_ are identical in value; A and B) or varies with increasing magnitude and trending upwards (panels C-F). Left-hand panels (A, C, E) show non-evolving systems with values of μ_mRT_ fixed. Right-hand panels (B, D, F) show the same system but with μ_mRT_ evolving according to GREM (Fig. 2). Values of SfG are also shown.

The different components of the GREM are described in the sub-sections below through reference to Fig. 2, but first, we outline the food web model used (Fig. 1). Table I describes variables mentioned in this main text; a Supplementary Table provides a concise description of all model parameters, together with their units, and also provides the parameter values used for the simulations of Figs 3–10. Reference to equations in the Supplementary Appendix use the style Equation (A*x*).

**Table I:** Variables mentioned in the main text and figures.

Variable	Unit	Variable type	Description
AE	dl	c	Assimilation efficiency of prey biomass by predator
CR_const_	dl	c	Catabolic respiration rate multiplier against μ_mT_; this is considered to increase in response to raised stress that elicits a metabolic up-shock, such as changes in salinity or pH
CR_exp_	dl	c	Stress-related multiplier upon the value of CR_const_
Cri	gN gN^−1^ d^−1^	a	Biomass-specific prey capture rate
Crit	mgN m^−3^ d^−1^	a	Value of [Crit (↓) − Crit (↑)] defining direction of evol
Crit (↓/↑)	mgN m^−3^ d^−1^	a	Net change in population biomass with ↓ or ↑evolving μ_mRT_
Dep	m	c	Mixed layer depth
Dil	d^−1^	c	Dilution rate
evol	d^−1^	a	Evolutionary increment for change of μ_mRT_, positive (↑) or negative (↓)
GR_pot_	d^−1^	a	Potential gross growth rate
Grow (↓/↑)	mgN m^−3^ d^−1^	a	Growth in population biomass with ↓ or ↑evolving μ_mRT_
Loss(↓/↑)	mgN m^−3^ d^−1^	a	Metabolic-related loss in population biomass with ↓ or ↑evolving μ_mRT_
Loss_reg_	d^−1^	a	Metabolic loss rate
mc	d^−1^	a	Stress-related mortality coefficient
M (↓/↑)	mgN m^−3^ d^−1^	a	Mortality in population biomass with ↓ or ↑evolving μ_mRT_
M_r_	d^−1^	a	Intrinsic mortality rate
N	mgN m^−3^	SV	Inorganic nutrient nitrogen
N_init_	mgN m^−3^	c	Initial and external N
Phyto	mgN m^−3^	SV	Phytoplankton biomass
Q_10_	dl	c	Multiple increase of μ_mRT_ per increase in T by 10°
RT	°C	c	Reference temperature
SfG	dl	a	SfG
T	°C	c	Contemporary temperature
T_dif_	°C	a	T minus reference temperature used to define μ_mRT_
Zoo1	mgN m^−3^	SV	Zooplankton(1) biomass
Zoo2	mgN m^−3^	SV	Zooplankton(2) biomass
μ_net_	d^−1^	a	Net growth rate
μ_m_	d^−1^	-	Maximum growth rate (in general terms)
μ_mRT_	d^−1^	SV	Value of μ_m_ at reference temperature, RT
μ_mT_	d^−1^	a	Value of μ_m_ at contemporary temperature, T
μ_T_	d^−1^	a	Emergent growth rate
λ	dl	c	Control constant for evol as Crit tends to zero

Variable types are: c, constant; a, auxiliary (emergent property); SV, state variable; ‘-’ general abbreviation, not used in equations; dl, dimensionless. See Supplementary Appendix for further information and values used in the different simulations.

### The food web model

To demonstrate the functioning of GREM, we used a simple nutrient-phytoplankton-zooplankton(1)-zooplankton(2) construct (hereafter, NPZZ) in which only a single element, nitrogen (with units of mg N m^−3^), was considered. This NPZ-type of construct has parallels in traditional marine ecosystem simulations (Fasham *et al*., 1990), of the type that forms the basis of most Earth systems models informing the Intergovernmental Panel on Climate Change (Arora *et al*., 2013). Details of the equations describing the plankton components, Phyto Zoo1 and Zoo2, as deployed here, are given in the Supplementary Appendix. The exact form of the descriptions for each evolving organism does not affect the use of GREM except with one important proviso: it is imperative that there are no inadvertent linkages between the evolving trait of the maximum growth rate and other trait descriptions. This matter is explained for our model as used in the Supplementary Appendix, with further general consideration in Discussion.

In brief, the NPZZ construct we used contains dissolved inorganic nitrogen (N) that supports the growth of the phytoplanktonic primary producer (Phyto), which is prey for a zooplanktonic secondary producer (Zoo1) and thence an additional zooplanktonic tertiary producer (Zoo2). Zoo1 not only grazes on Phyto but also cannibalizes itself, whereas Zoo2 not only grazes on Zoo1 but cannibalizes itself (Fig. 1). The plankton may be considered as functional types (with simulations describing mean activity), or as named species; we make no differentiation between these options here. Note that from hereon, the terms ‘Phyto’, ‘Zoo1’ and ‘Zoo2’ describe both the names of the plankton and the state variables representing their biomass concentrations in the simulations.

The system is, as a real system, not sealed but is subjected to a low-level dilution rate (0.05 d^−1^, akin to a mixing rate across an ergocline) that brings in N, and washes out residual N and also a proportion of Phyto, Zoo1 and Zoo2 (none of which are assumed to be capable of swimming against such a mixing rate). Surface irradiance (Photon Flux Density) is described as varying on a 12 h light (PFD = 1 000 μmol photons m^−2^ s^−1^) and 12 h dark (PFD = 0) cycle. The model food web was operated in a simulated water column of fixed mixed depth (Dep) and nutrient loading (N_init_). Dep and N_init_ interact to light-nutrient limit the rate of production.

### The growth rate evolution model

The conceptual structure of GREM is shown in Fig. 2A, with a flow chart for its operation in Fig. 2B. In essence, and as described in detail below, the maximum growth rate for each organism (as stated at a reference temperature; termed μ_mRT_) evolves to balance the advantage of possessing a high μ_m_ against the metabolic cost incurred to maintain this trait during growth in the current environmental setting. A fundamental feature of GREM is that μ_mRT_ for each plankton component is defined by a state variable whose value can evolve to higher or lower values during the simulations. All variables mentioned in the text below are defined in Table I.

### Net growth rate and the effect of temperature in the GREM model

Because the rate of biological processes varies with temperature, we differentiate between the value of μ_m_ at reference temperature RT (defined as μ_mRT_) and the value at the contemporary temperature in the simulation (μ_mT_). It is the value of μ_mRT_ that evolves; μ_mT_ differs from μ_mRT_ by a factor reflecting an environmental effect, which has no genetic basis upon which to evolve. Initial values of μ_mRT_ (ca. 1 d^−1^) were selected in accordance for small plankton species.

Assuming the range of T is not extreme, we define μ_mT_ using an Arrhenius function with reference to a value of Q_10_ (where Q_10_ defines the multiple increases of growth rate per increase in T by 10°; e.g. Q_10_ = 2 doubles the rate over a 10°C increase in T). Because we only need the difference between RT and T, here we refer to that difference (T_dif_ = T-RT). Thus:

(1) \begin{equation*} {\mu}_{\mathrm{mT}}={\mu}_{\mathrm{mRT}}\times{\mathrm{Q}}_{10}^{\left(\frac{{\mathrm{T}}_{\mathrm{dif}}}{10}\right)} \end{equation*}

At the reference temperate if T_dif_ = 0, μ_mT_ = μ_mRT_.

The net specific growth rate (μ_net_) is a function (Equation 2) of the potential gross growth rate (GR_pot_), the metabolic loss rate (e.g. catabolic and anabolic respiration rates, which may be directly related to stress such as to changes in pH with ocean acidification (OA), and are associated with N regeneration N; Loss_reg_), and an intrinsic mortality rate (M_r_), which is also expected to increase under stressful conditions.

(2) \begin{equation*} {\mu}_{\mathrm{net}}={\mathrm{GR}}_{\mathrm{pot}}-{\mathrm{Loss}}_{\mathrm{r}\mathrm{eg}}-{\mathrm{M}}_{\mathrm{r}} \end{equation*}

In the Supplementary Appendix, we explain how the terms on the RHS of this equation are derived for our specific model, recognizing that a variety of assumptions are made in the literature in the construction of NPZ-type models and set against the caveat noted above concerning the importance of avoiding any inadvertent linkage of other traits to the evolution of μ_mRT_.

### Scope for growth

The values of GR_pot_ (d^−1^) and Loss_reg_ (d^−1^) for each organism type, as described in the Supplementary Appendix, are incorporated in an index that represents the proportion of potential specific growth rate, which contributes to net growth rate. We call this ‘scope for growth’ (SfG; dimensionless):

(3) \begin{equation*} \mathrm{SfG}=\left({\mathrm{GR}}_{\mathrm{pot}}-{\mathrm{Loss}}_{\mathrm{reg}}\right)/{\mathrm{GR}}_{\mathrm{pot}} \end{equation*}

The specific definitions as applied to Phyto, Zoo1 and Zoo2 are given in the Supplementary Appendix.

A decrease in SfG, resulting from an increase in Loss_reg_ relative to GR_pot_, may be considered as an indication of stress requiring mitigating responses by the organism; a failure or inability to moderate this stress can be interpreted as a failure of homeostatic mechanisms. In the model, the stress associated with a decrease in SfG causes a proportional additional mortality loss to the organism (Supplementary Appendix). The net result is that SfG is optimized concurrently with the evolution μ_mRT_ at an equilibrium value (Fig. 2), as functions of the prevailing conditions (e.g. mixed layer depth, light, temperature, and nutrient load) and parameter values describing physiology.

Physiological processes change continuously, whereas organisms integrate those processes and associated losses over time spans that relate to the life cycle duration of the organism. In the model, Phyto, Zoo1, Zoo2, μ_mT_ and SfG may change at every timestep; to rationalize model behaviour with reality, we operationally define SfG as a rolling average over 1 day.

### Evolution of μ_mRT_

GREM enables the value of μ_mRT_ for each plankton component to evolve to higher or lower values during the simulation by discrete amounts at each timestep. We justify this approach by the fact that plankton populations have substantial genetic variation on which selection can act (Reusch and Boyd, 2013; Collins *et al*., 2014; Bach *et al*., 2018), which allows a quantitative genetics approach without the need to specify gene frequencies for the traits of interest. In this respect, the model is similar to adaptive dynamics approaches (Abrams, 2005; Waxman and Gavrilets, 2005). The evolutionary model does not follow the fate of rare mutations invading resident populations. Instead, it follows the changes in the value of μ_mRT_ as a consequence of the fitness effects of small increases or decreases in the trait, as explained below.

In general, in a quantitative genetics model the response to selection can be represented by an expression such as *R* = *i* × *h*^2^ × *x_p_* where *R* is response to selection, *i* is the intensity of selection, *h*^2^ is the heritability of the character, and *x_p_* is phenotypic standard deviation of the trait. Values for the independent variables in this equation are not well known for most populations, including plankton. We therefore employ a heuristic approach to determine the direction of change of μ_mRT_, as described below, following the schematic in Fig. 2B.

Variable evol defines the amount that μ_mRT_ either increases or decreases at each timestep in the simulations. The method of computation of evol is described in the Supplementary Appendix. For Phyto, as example, the net change in biomass is calculated prospectively for two scenarios in which ${\mu}_{\mathsf{mRT}}^{\mathsf{Phyto}}$ is increased (↑) or decreased (↓) by an amount evol^Phyto^, thus:

(4) \begin{equation*} {\mu}_{\mathrm{mRT}}^{\mathrm{Phyto}}\left(\uparrow \right)={\mu}_{\mathrm{mRT}}^{\mathrm{Phyto}}+{\mathrm{evol}}^{\mathrm{Phyto}} \end{equation*}

and

(5) \begin{equation*} {\mu}_{\mathrm{mRT}}^{\mathrm{Phyto}}\left(\downarrow \right)={\mu}_{\mathrm{mRT}}^{\mathrm{Phyto}}-{\mathrm{evol}}^{\mathrm{Phyto}} \end{equation*}

As the value of ${\mu}_{\mathsf{mRT}}^{\mathsf{Phyto}}$ is held in a state variable, operationally ±evol represents a timescale dependant differential (thus ±evol × dt).

From these options (Equation (4) vs Equation (5)), critical values representing net change in population biomass based on Equation (2) (see Supplementary Appendix for this specific model implementation, Equations (A6), (A17) and (A23)) are defined in general terms as:

(6) \begin{equation*} {\mathrm{Crit}}^{\mathrm{Phyto}}\left(\uparrow \right)={\mathrm{Grow}}^{\mathrm{Phyto}}\left(\uparrow \right)-{\mathrm{Loss}}^{\mathrm{Phyto}}\left(\uparrow \right)-{\mathrm{M}}^{\mathrm{Phyto}}\left(\uparrow \right), \end{equation*}

which is calculated employing ${\mu}_{\mathsf{mRT}}^{\mathsf{Phyto}}$ (↑), and

(7) \begin{equation*} {\mathrm{Crit}}^{\mathrm{Phyto}}\left(\downarrow \right)={\mathrm{Grow}}^{\mathrm{Phyto}}\left(\downarrow \right)-{\mathrm{Loss}}^{\mathrm{Phyto}}\left(\downarrow \right)-{\mathrm{M}}^{\mathrm{Phyto}}\left(\downarrow \right), \end{equation*}

which is calculated employing ${\mu}_{\mathsf{mRT}}^{\mathrm{Phyto}}$ (↓).

A single critical value is then defined as:

(8) \begin{equation*} {\mathrm{Crit}}^{\mathrm{Phyto}}={\mathrm{Crit}}^{\mathrm{Phyto}}\left(\uparrow \right)-{\mathrm{Crit}}^{\mathrm{Phyto}}\left(\downarrow \right) \end{equation*}

If Crit^Phyto^ > 0, the simulation will proceed via the scenario which has ${\mu}_{\mathsf{mRT}}^{\mathsf{Phyto}}$ increased by evol^Phyto^; if Crit^Phyto^ < 0, it will proceed via the scenario with ${\mu}_{\mathsf{mRT}}^{\mathsf{Phyto}}$ decreased by evol^Phyto^.

Thus, the prospective analysis sets the direction of the change according to whether an increase or decrease in ${\mu}_{\mathsf{mRT}}^{\mathsf{Phyto}}$ would be favoured by directional selection. If Crit^Phyto^ = 0, ${\mu}_{\mathsf{mRT}}^{\mathsf{Phyto}}$ will not change. The state variable ${\mu}_{\mathsf{mRT}}^{\mathsf{Phyto}}$ is thus either increased or decreased by the amount evol^Phyto^. The new value of ${\mu}_{\mathsf{mRT}}^{\mathsf{Phyto}}$ is then used to calculate the operational value of ${\mu}_{\mathsf{mT}}^{\mathsf{Phyto}}$ for the next timestep according to Equation (1).

Over time ${\mu}_{\mathsf{mRT}}^{\mathsf{Phyto}}$ approaches, an equilibrium value at which point the value of Crit^Phyto^ tends to zero. Crit^Phyto^ can thus be regarded as analogous to a fitness function, which is optimized at the value 0. As Crit^Phyto^ approaches zero, the selection pressure is expected to become weaker and the value of evol^Phyto^ would thus be expected to decrease. This has been simulated by allowing the value of evol^Phyto^ to decline towards zero as equilibrium is approached (Supplementary Appendix).

**Table II:** Summary of simulations carried out. Variables are defined in the text and Table I.

Type of simulation	Summary	Figures
Phyto chemostat	150 factorial simulations	Figs. 3 and 4
Time course	CR_const_ changes	Fig. 5A
Time course	Dep changes	Fig. 5B
Time course	evol lower and higher; mc lower	Fig. 6A
Time course	evol lower and higher; mc higher	Fig. 6B
NPZZ	200 simulations over parameter space	Figs. 7 and 8
Time course	T_dif_ changes abruptly	Fig. 9
Time course	CR_const_ fluctuates	Fig. 10A and B
Time course	T_dif_ fluctuates	Fig. 10C and D
Time course	CR_const_ and T_dif_ fluctuate	Fig. 10E and F

See Supplementary Table for details of parameter values.

The same type of model construct is used for Zoo1 and Zoo2, computing evol^Zoo1^ and evol^Zoo2^, with net change in plankton biomass defined by the grazing (Supplementary Appendix Equations (A15) and (A16)) less the metabolic losses (Equation (A18)) and losses as a function of SfG (Equations (A24) and (A25)). The formulation of Crit for each component excludes from the losses those that the component incurs by being consumed by grazing by a higher predator. This is because this predation is, in the context of this particular model, an external factor beyond the direct influence of the prey and not expected to cause selective mortality influencing evolution of μ_mRT_ in the prey. For consistency cannibalism is similarly excluded.

The model was also run for Phyto in a chemostat-style scenario without Zoo1 and Zoo2, and in which the dilution rate forces the net growth rate (i.e. dilution rate = ${\mu}_{\mathrm{net}}^{\mathsf{Phyto}}$), and growth was constrained by the residual abundance of the limiting resource N. The simulations were run at the reference temperature, thus T_dif_ = 0 and hence ${\mu}_{\mathsf{mT}}^{\mathsf{Phyto}}$ = ${\mu}_{\mathsf{m}\mathrm{R}\mathsf{T}}^{\mathsf{Phyto}}$. Respiratory losses associated with ${\mathrm{CR}}_{\mathrm{const}}^{\mathrm{Phyto}}$ were allowed to regenerate N. However, the SfG^Phyto^ dependent mortality of Phyto was not recycled back into N on the assumption that degradation of corpses would not occur at a significant rate within the chemostat. Instead, this mortality loss of Phyto flowed out of the chemostat to a sink.

The model was implemented in Powersim Studio 10 (www.powersim.com), which runs simulations using ordinary differential equations, here using an Euler integration routine. Simulations were run over 2000 or 3000 days; in constant conditions, equilibrium was usually reached by 500 days or earlier. Statistical analysis was carried out with Statistical Package for the Social Sciences (IBM SPSS Statistics).

## RESULTS

Table II summarizes the simulations carried out and the figures in which the simulation results are presented.

Figure 3 shows simulations where μ_mRT_ evolves to equilibrium for Phyto growing in a chemostat-type environment (where μ_net_ is set by the dilution rate, Dil; d^−1^). Figure 3A shows that equilibrium values of μ_mRT_ decrease with decreasing Dil and thence with enforced nutrient-limited μ_net_ (consistent with (Droop, 1974)). The actual relationship differs with different levels of catabolic respiration that imparts additional stress. Higher μ_mRT_ -dependent stress, imparted here by an elevated catabolic rate using higher values of the parameters CR_const_ and CR_exp_, leads to the evolution of a lower μ_mRT_. SfG shows comparatively uniform values across the parameter ranges. Residual N is somewhat increased by increasing values of all parameters to ensure that net growth matches Dil in the face of increased catabolic loss and SfG dependent mortality. At the maximum value of all parameters (bottom right mesh in Panel C), a higher-order interaction is evident as the surface curves upwards to a higher residual N value (Fig. 3C).

The standardized β values of the regression statistics for the Phyto chemostat (Fig. 4) confirm the visual interpretation from the mesh plots. Thus, μ_mRT_ is positively associated with Dil but is decreased by higher catabolic losses (negative β values for CR_const_ and CR_exp_), and residual N is positively associated with all parameters. More evident than in the mesh plots, which shows unstandardized values, is that SfG is depressed by higher values of CR_const_ (i.e. by stress) but increases slightly with higher value of mc which influences SfG dependent mortality. Overall adjusted R^2^ values for main effects for μ_mRT_, SfG, and N are 0.875, 0.842 and 0.699, respectively. At the maximum value of all parameters (bottom right mesh in Fig. 3C), a higher-order interaction is evident as the surface curves upwards markedly to a residual N value which is clearly higher than would be expected due to additive effects of the three parameters (Fig. 3C). This is indicative of a synergistic response to multi-stressors.

Operating GREM within the dynamic food web NPZZ model shows that the pattern of evolution of μ_mRT_ and SfG is perfectly reversible under the relief of different stressors (Fig. 5). Initial biomasses of the components affect the dynamics but not the final equilibrium values at steady state (not shown). The rate of evolution towards the equilibrium value can be varied using the mortality parameter mc and also a parameter λ that affects the magnitude of evol (see Supplementary Appendix). A higher mortality results in a more rapid approach to the equilibrium μ_mRT_ and also lower equilibrium values for all three plankton components (mc = 1, Fig. 6B compared with mc = 0, Fig. 6A). The order of values of μ_mRT_ for the different trophic levels remains the same, in reverse order of the trophic level. A lower value of evol results in a less rapid approach to the equilibrium value of μ_mRT_ (λ = 1, dashed lines compared with λ = 0.5, solid lines). However, the equilibrium values are not affected by this difference in evolutionary rate as dashed and solid lines converge.

Results from an analysis of 200 simulations of the NPZZ model, run to equilibrium under different conditions, are provided in Fig. 7. In Fig. 7A, the mean values are compared in those of the simulations where Zoo2 is lost and those where it is retained at equilibrium. Loss is thus indicative of system stress leading to low relative fitness of Zoo2 and its subsequent extinction of this organism from the system. Higher values of CR_const_ are associated with this stress, by analogy with the relationship observed in the Phyto chemostat (Fig. 3). Also associated with higher stress leading to Zoo2 extinction are lower values of assimilation efficiency (AE) and greater water depth (Dep) that restricts the entry of energy and thus primary production to the system; these results are consistent with expectations. Smaller effects associated with Zoo2 loss, but also in line with expectation, are higher SfG stress-dependent mortality (mc), lower capture rate (Cri) and lower initial N in the simulation. In Fig. 7B, the β values for linear regression analysis are shown to allow comparison of the parameters for their quantitative and direction of effects on μ_mRT_. Higher values of CR_const_ are seen to favour the evolution of lower values of μ_mRT_ (large negative β). Since from Fig. 7A, we observe that higher CR_const_ equates to higher stress, cross comparing with Fig. 7B we can conclude that this higher stress is also associated with lower values of μ_mRT_. Using similar reasoning, higher AE, and shallower water depth (lower Dep, and thence increasing light energy availability and higher primary production), are also associated with lower stress and higher μ_mRT_. The parameters Cri, mc and N_init_ have low β values and are thus less influential in explaining variation in μ_mRT_, though of these higher Cri are associated with higher μ_mRT_. The approach of comparing Fig 7A and B thus allows confirmation of *a priori* expectations of the effects of parameters in relation to stress and overall, these expectations are confirmed well. Using similar reasoning for explaining Fig. 7C, lower CR_const_, higher AE (for Zoo1 and Zoo2) and shallower depth (for Phyto) are associated with lower stress and higher values of SfG, again in line with expectation. Overall adjusted R^2^ values for main effects for μ_mRT_ for Phyto, Zoo1 and Zoo2 are 0.832, 0.747 and 0.737, respectively increasing to 0.851, 0.825 and 0.853 when first order interactions are included. For SfG, the corresponding groups of values for main effects are even higher at 0.958, 0.996 and 0.996. The variation not captured will be due to non-linearity and higher-order interactions, but it is clear that the parameter main effects have substantial explanatory power.

The simulations of Fig. 7 were repeated with identical parameters but with evolution disabled, by setting evol for each of Phyto, Zoo1 and Zoo2 to zero, in order to demonstrate the added effect on Zoo2 when moving from a scenario with no evolution to one with evolution (Fig. 8). With no evolution, Zoo2 survives in 76 out of the 200 simulations, whereas with evolution Zoo2 survives in 135 simulations, providing evidence of evolutionary rescue. In the majority of evolving simulations, the biomass of Zoo2 is higher (i.e. normalized (evol-no evol) > 0, Y-axis), with most plotted points > 0; this indicates that evolution favours the production of a higher biomass. Thus, on average over all the 200 simulations, the biomass of Zoo2 is 38% higher with evolution, and in the 76 simulations in which Zoo2 survives with and without evolution, the biomass of Zoo2 is 24% higher with evolution.

In a system where temperature increases abruptly and then falls abruptly, clear differences are seen between non-evolving and evolving systems (Fig. 9). Initially T_dif_ is set at 0 (i.e. T = RT, so μ_mT_ and μ_mRT_ are equal). In the non-evolving system, in which μ_mRT_ remains constant, μ_mT_ increases to a new stable value as the temperature rises. For Phyto, SfG declines, although the biomass of Phyto increases slightly (Fig. 9A), likely reflecting consequences of trophic interactions. SfG and biomass for Zoo1 and Zoo2 are little affected by the temperature increase. On reversal of the temperature change, the original values of μ_mT_, SfG and biomass are restored. In the evolving system, initially, the same response to the increase in temperature is seen as in the non-evolving system. However, μ_mRT_ then evolves downwards so that the expressed μ_mT_ for all components tend towards the equilibrium values expressed at the lower original temperature (Fig. 9B); these were the operational optimal values for μ_mT_ and μ_mRT_ given the other parameter values used in the simulation. SfG for Phyto initially shows a small decline as the temperature increases but then evolves towards its original value. When temperature is decreased, the system, and its evolving characters revert to their original values.

In Fig. 10 are shown, for both non-evolving and evolving systems, changes caused by an oscillating stress (CR_const_) that raises respiration, and/or changing temperature (T_dif_). Under conditions of increasing oscillations in degree and frequency of stress alone (Fig. 10A and B), varying patterns emerge in the evolution of μ_mRT_, consequential changes in SfG, and in organism biomass. Biomass oscillations are markedly different with evolution (Fig. 10B) compared to food webs without evolving capabilities (Fig. 10A). With evolution, values of μ_mRT_ decline in consequence of increasing stress, and there are less marked fluctuations in SfG and biomass in comparison with non-evolving simulations. With upward-trending temperatures (Fig. 10C and D), μ_mT_ fluctuates in synchrony with the fluctuating temperature. In the evolving system (Fig. 10D), μ_mRT_ evolves downwards as temperature trends upwards overall; this results in μ_mT_ moving down towards a lower equilibrium value as the average temperature rises. This is most evident for Phyto and is consistent with Fig. 9B where the effects of longer-term stable changes in T_dif_ are shown. There are no noticeable differences in SfG between the non-evolving and evolving scenarios, and the period of the temperature oscillations is too short to allow new equilibrium levels to be discernible.

With oscillations in both temperature (T_dif_) and in environmental stress (enacted by changing CR_const_), there are more significant differences between non-evolving (Fig. 10E) and evolving (Fig. 10F) systems. Note that here Zoo2 becomes extinct at around 1300 days in the non-evolving system (Fig. 10E). As seen before in evolving systems (Fig. 8), evolution allows for the potential of evolutionary rescue; here, in the evolving system (Fig. 10F), there are less marked fluctuations in biomass when evolution is allowed, and evolution also protects better against low SfG and low component biomass values—extinction is less likely and here Zoo2 survives (Cf. Fig. 10E).

## DISCUSSION

Evolution is inseparable from ecology, yet in simulations of plankton ecology that extend over what amounts to many 100s if not 1000s of generations, this critical process has received little attention. Here, we have presented a demonstrator for an approach to simulating evolution, together with illustrations of its operation to indicate some implications for simulating long-term plankton dynamics without or with evolution. We discuss below some of the features of this development.

Investigations of processes that are consequences of evolution have been widely explored through trait-trade-off (TTO) approaches. Allometric-linked aspects of phytoplankton nutrient acquisition (e.g. Litchman *et al*., 2006, 2007; Finkel *et al*., 2010; Andersen *et al*., 2016) have provided a rich source of inspiration for many modellers. Such concepts build from the classic view that the half-saturation constant for resource acquisition is a pivotal trait defining competition advantage (Tilman, 1976; Morel, 1987) and that smaller organisms may be expected to exhibit lower half saturation values and grow faster. However, various questions can be raised over the interpretation of experimentally derived half-saturation constants for nutrient uptake (Flynn *et al.*, 2018), and also whether analogous mathematical constructs for food (prey) acquisition are even appropriate (Flynn and Mitra, 2016).

Perhaps the best-known deployment of TTO concepts at a grand scale has been the DARWIN model of Follows *et al*. (2007), in which a wide range of phytoplankton functional type descriptions compete in an *in silico* ocean. Such modelling efforts, however, do not describe or consider evolutionary processes during the simulation and depend upon a series of fixed assumptions for TTOs. There is no universal TTO considered in such models except perhaps that small cells generally have the potential to grow faster. That relationship is at best described as a scatter plot (e.g. Fig. 3 in Finkel *et al*., 2010), and we must conclude that there must be a set of higher-level factors that control the potential for growth in real populations (Boyd *et al*., 2013). What our work demonstrates is that through evolution we should expect significant variation in expression of the maximum growth rate by otherwise similar organisms growing in different environments. We also expect those higher-level factors to include resource acquisition and trophic interactions acting throughout the food web.

Locating traits that are of universal importance or are of sufficient commonality, to justify modelling their evolution is far from simple. Developing evolutionary simulations also rather forces the modeller to revisit other parts of the model, to ensure there are no unintended consequences of the *in silico* evolution of the selected trait(s). While the basis of the approach we describe for GREM also provides a tractable tool for exploring the evolution of other traits, there is an important general proviso that the evolving traits must not confound the description of other traits in unrealistic ways. The models must also not be constructed in such a way that permits the evolution of traits to evolve to ever higher or unrealistic values (such as the maximum rate of C-fixation linked to RuBisCO activity (Flynn and Raven, 2017). So, in the context of GREM, it is imperative that for no organism sub-model is there an inadvertent linkage between the evolving trait of μ_mRT_ and other trait descriptions.

Here, through Equation (A4), we overcame such a challenge which would otherwise have seen an evolutionary increase in μ_mRT_ occurring simultaneously with an enhanced nutrient acquisition potential. This comes about because retention of the same half-saturation constant for resource acquisition (K_g_) with an increasing μ_mRT_, inadvertently provides the simulated organism with an additional advantage as the growth rate at limiting nutrient concentrations is also raised. However, such an event is implausible because unless the resource acquisition kinetics (i.e. nutrient transportation system) also evolved simultaneously, then a slower-growing variant should exhibit a lower K_g_ and thus be at a competitive advantage (Flynn *et al*., 2018) in low-nutrient system, as expected of slower growing ‘K-select’ species (Tilman, 1976). An allied issue is the importance of using an appropriate consumer (zooplankton) description. Often feeding (grazing) descriptions use an approach where the zooplankton maximum growth rate is modelled as an emergent function of feeding as restrained by a stated fixed maximum feeding rate (Gentleman *et al*., 2003; Mitra *et al*., 2014a; a so-called ‘live-to-eat’ approach as termed by Flynn, 2018). Using this approach, irrespective of food availability, the simulated consumer can never respond to stress by increasing its feeding rate. Here we used an ‘eat-to-live’ functionality (Flynn, 2018), in which the maximum feeding rate is a function of the maximum growth rate. This description enables the simulated zooplankton to increase its potential to feed in response to an increased demand for energy to compensate for elevated stress, as is expected in reality. This ‘eat-to-live’ implementation, together with an encounter-based grazing function (Mitra and Flynn, 2006; Flynn and Mitra, 2016), enables the μ_mRT_ for each consumer zooplankton to usefully evolve in a model.

In more general terms our model simulates what we propose to be a universal TTO, namely balancing the advantage of processing a high potential growth rate against the disadvantage of the stress of being unable to fulfil that potential due to external factors. Flynn (2009) suggested a simple empirical approach to this topic, working on the argument that maintaining a good state of organism health (low stress) is important to survival. This was inspired by Droop’s (1974) observation that a phytoplankton culture forced to grow slowly in a nutrient-limited chemostat lost the ability to grow at a high rate; over the course of several months, the organisms evolved to have a lower maximum growth rate. Of course, the allometric-linked TTO relationships mentioned above make an individual organism more or less able to compete well in nutrient-stressed conditions, but the central tenet in the work developed in the current paper is that all those very many traits and their putative TTOs find a focus in supporting the potential for delivery of a growth rate closer to, rather than further from, a given maximum growth rate potential. A TTO reflected in our current results, seen in the simulations carried out over parameter space (Figs. 7 and 8), is the contrast between higher maximum growth with higher respiratory costs and lower maximum growth rate with lower respiratory costs, the two scenarios being associated with different sets of parameter values but giving the same value of SfG.

Higher growth rates, if sustainable, are of clear competitive advantage for population growth, whereas increasing costs associated with high growth rate when conditions are less favourable leads to increased stress and mortality. There are various lines of supporting evidence for the evolution of μ_mRT_ to represent a universal TTO across many realms of biology and ecology. During growth, fewer resources might be allocated to energy reserves, which may in turn lead to vulnerability to stress and mortality (Dmitriew, 2011). Rapid growth may also lead to cellular damage and mortality as a result of free radical generation and is expected to be proportionally greater at higher growth as result of progressive damage to mitochondrial membranes (Mangel and Munch, 2005; Monaghan *et al*., 2009). In yeast, the death rate per generation has been observed to increase with the division rate (Nakaoka and Wakamoto, 2017). In *Escherichia coli*, the sensitivity to stress of mild heat, ultra violet type A light and sunlight is greater for cells with higher specific growth rate (Berney *et al*., 2006). In zebra finches, free radical damage to red blood cells is correlated with growth rate, suggesting that oxidative damage may constrain growth rate (Alonso-Alvarez *et al*., 2007). Standard metabolic rate is higher for a fast-growth genotypes in the fish *Menidia* (Arnott *et al*., 2006) and rainbow trout (Allen *et al*., 2016). Finally, a variety of maintenance costs such as metabolic shifts, molecular turnover and defence against stress, also subtract from the intrinsic growth rate and show a clear positive correlation with maximum growth rate potential across a range of microbial species (van Bodegom, 2007). All such data point to a distinct risk, of additional costs, to be able to grow fast, setting a trade-off that is only worth exploiting when the potential is realizable.

GREM is a deterministic model for evolution in line with observations that plankton populations are large, providing substantial genetic variation on which selection can act to cause phenotypic shifts (Reusch and Boyd, 2013). In this circumstance, the standing genetic variance can be assumed to be ever present (Jiang *et al*., 2005). We thus use a quantitative genetics approach without specifying gene frequencies and in which adaptation is not mutation limited. The approach allows evolutionary equilibria as a function of parameter values to be determined easily and quickly. This makes feasible running large numbers of simulations across parameter space where each is run up to several thousand days to equilibrium. Given initial parameter values, our simulation results are repeatable and reversible (Fig. 5), consistent with the view that evolution is often repeatable given similar environmental conditions (Blount *et al*., 2018). Of course, in nature, such conditions are most unlikely to be met due to the complexity of the biotic and abiotic interconnectivities and feedbacks, and alternative steady-state solutions may exist. Oscillatory and chaotic behaviour occurs in predator-prey systems including NPZ constructs in some circumstances (Edwards and Brindley, 1996; Sherratt *et al.*, 1997; Gibson *et al.*, 2005). As can be seen in many of the simulations presented here, there are no noticeable oscillations in biomass at equilibrium that facilitates the attributing of the variation in final equilibrium values to variation in parameter values. Results with evolution are nevertheless highly contingent on the initial values of the parameters (conditions) we use and are thus consistent with the divergent outcomes observed in nature and in laboratory experiments. We demonstrate a regression method to assess the quantitative importance of the parameters in explaining the variation in output variables in samples of simulations across parameter space both for the Phyto chemostat (Fig. 4) and the NPZZ system (Fig. 7). This method may be useful generally in analysis the results of NPZ type simulations. The role of interactions between multi-stressor traits is potentially of great importance in plankton communities, requiring further study (Taherzadeh *et al.*, 2019) In our results, parameter main effects are dominant quantitatively but a higher-order interaction was clearly evident in the Phyto chemostat indicating synergism in the response to multi-stressors (Fig. 3).

A consistent and expected feature of the results is that higher trophic levels have lower biomass at equilibrium (for example, Figs 9 and 10). More interesting is that maximum growth rate and SfG also follow this trend as an emergent property of GREM (Figs 5, 6, 10 and 11). Equilibrium values are usually reached in the simulations in a few hundred days (for example, Figs 5 and 6) but the rate of approach can be affected by parameter values. For example, higher mortality indicating more intense selection, or increase in the size of the evolutionary increments set by the variable evol increases the rate of approach to equilibrium (Fig. 6). Equilibrium values in the simulations attained over 100 s of days are consistent with experimental evolution in plankton populations where genetic adaptation to stressors occur on similar timescales, much shorter than that for ongoing climate change (Bach *et al*., 2018).

The results shown in Fig. 7 provide an insight as to whether high or low parameter values are associated with stress as judged by Zoo2 loss (Fig. 7A), and thence whether this stress is associated with higher or lower values of μ_mRT_ (Fig. 7B). We can see that higher basal respiration (CR_const_), lower AE, and lower rates of energy input (higher value of Dep, restraining primary production) are all associated with higher stress and lowering of μ_mRT_. The direction of evolution described by GREM is entirely consistent with intuitive expectations of the effect of higher or lower values of these parameters.

In total then, the behaviour of the model running with GREM describing evolution is consistent with expectations. The GREM approach allows us to tentatively explore the implications of climate change on evolution and ecology in different model systems in future research. Because of its fundamental importance for adaptation and survival, evolutionary change needs to be more extensively represented in models of climate change. Our results show that when the maximum growth rate of an organism is allowed to evolve, as occurs in the real world, resource allocations and acquisitions show different dynamics to when such evolution is disallowed; these changes are reflected in changes in population dynamics and indeed in the evolution of μ_mRT_ and thence in the expression of μ_T_. We must then also expect that organisms growing in mature ecosystems (K-type, e.g. in the temperate summer) will have lower μ_mRT_ than those growing in immature ecosystems (r-type organisms, e.g. in the spring bloom). This can then also explain why mixoplankton, which one may otherwise expect to be able to grow rapidly by mixotrophically exploiting various nutrient resources but which also live in mature ecosystems (Mitra *et al*., 2014b), actually grow rather slowly compared with many non-mixoplankton isolates from immature ecosystems (Flynn *et al*., 2019).

Evolution in response to stress and temperature changes will be affected by changes in the whole ecosystem as there is adaptation to new conditions (Figs. 9 and 10). This is in line with the suggestion that complex models with competing species may give more realistic responses of species to climate change (Bach *et al*., 2018). The increased sensitivity of populations in the absence of evolution of μ_mRT_ (Figs. 8 and 10) implies also that small real populations, lacking genetic diversity, are also more likely to become extinct. In a system not impacted by frequent and/or severe stresses, μ_mRT_ evolves upwards, with elevated SfG, lowering the likelihood of extinction, and stabilizing trophic dynamics. Conversely, in an environment where conditions are severe more frequently, as expected under climate change (Doney *et al*., 2012; Thornton *et al*., 2014), μ_mRT_ evolves downwards (Figs. 5, 9 and 10B and F); evolution under climate change would then decrease production but enhance the prevention of extinction through evolutionary rescue (Bell, 2013; DeLong and Belmaker, 2019).

Interestingly, our simulations suggest that advantages for growth brought about by elevated temperatures (Toseland *et al*., 2013) may be countered at least in part by the evolution of decreased μ_mRT_ such that μ_mT_ is ultimately little changed (Fig. 10D); this will be so unless there are concurrent changes in factors that decrease stress so allowing an increase in μ_mRT_ that *de facto* exploits an improved SfG. Growing at higher temperatures raises the catalytic efficiency of enzymes and will thus alter resource allocations, unless thermal tolerance is approached, and costs or maintenance exceed gains. Here, we considered a generic stress that raises catabolic respiration and otherwise decreases SfG; for marine plankton, an example of such a stressor is OA. Responses to temperature changes and OA are widely acknowledged as being multi-stressors for marine organisms (Sommer *et al*., 2015), to which we can add the classically considered issues of nutrient, light availability and mixed layer depth; all these factors are expected to affect SfG in reality.

There are other features of organisms that are affected by growth rate, and hence that will impact upon competitive advantage and evolution, that we do not explore in our model but should be borne in mind. Some examples include the following: Organisms growing at different temperatures or under different types of nutrient stress often display different allometric responses (e.g. organisms may be larger when growing at low temperatures, affecting resource acquisition; see also Finkel *et al*., 2010). Behavioural responses of starved animals may expose them to different risks of predation. Such features can be included in the next explorations of the application of GREM—here we wished to demonstrate the concepts in a comprehensive way using an otherwise simple model.

The work we have undertaken raises many additional questions concerning evolution and modelling thereof. Is there much to gain from simulating the evolution of μ_mRT_ using an individual-based-model approach, or a multispecies consortium rather than functional type descriptions? How are dynamics affected by evolution in organisms that enter some form of stasis (encystment) under deleterious conditions? How important is the relative rate of evolution of different components in the food web? On the flip side, one may question whether explicitly modelling evolution is more useful than extending the DARWIN (Follows *et al*., 2007) approach and just supply a very large number of configurations for each functional types (including predators and now also mixoplankton; Flynn *et al.*, 2019). By allowing each plankton functional type to evolve, a computationally compact approach such as GREM may be cheaper to run in prolonged simulations than simulating many different functional type descriptions each differing in their fixed maximum growth rate. Understanding better how stress and satisfying organism’s demand for resources affects their ecology and thence ecosystem dynamics as each organism group evolves is also likely to be an interesting exercise.

To operate GREM within a simulation framework that requires the transference of material between adjacent grid cells, such as in 3D models, some thoughts are required for how to best represent the state variable describing μ_mRT_. In such models, it is necessary to define state variables in terms of mass (e.g. mgN m^−3^); a state variable with units of d^−1^ cannot be exchanged (shared) between grid cells. However, the value of μ_mRT_ represents a metabolic capacity, analogous to an enzyme activity (i.e. the k_cat_ value, with units of mole substrate per mol enzyme per second). As such the rate documented by μ_mRT_ is associated with a mass of biochemical components and could equally well be associated with a concentration of biochemical material (e.g. with units of mgN m^−3^), where each unit of that component is associated with an activity potential residing within an organism (which is assigned a state variable with mass concentration units, as usual).

## CONCLUSION

We conclude that it is as important to consider the evolution of μ_mRT_ in models as it is to include descriptions of competing species to provide more realistic simulated responses of ecology to climate change (Bach *et al*., 2018). GREM is computationally inexpensive to operate, requiring only one additional state variable for the evolving μ_mRT_ of each simulated organism. Although explored here for plankton dynamics, we suggest that conceptual underpinnings of GREM, which μ_mRT_ evolves to allow an organism to match its demands for resources against the supply, is likely to be universal across all life forms. As such GREM provides a useful tool to explore evolution affecting individual species, ecosystems and biogeochemical processes at all levels.

### DATA ACCESSIBILITY

All data and model equations are available in the main text or the Supplementary Appendix or Supplementary Table.

## AUTHORS’ CONTRIBUTIONS

D.O.F.S .built and ran the GREM model from an original idea and modelling concept of K.J.F. Authors equally shared the setting of simulation scenarios, interpretation of results, and writing of the paper.

## COMPETING INTERESTS

We have no competing interests.

## Supplementary Material

Flynn_Skibinski_Supplementary_Table_rev_July_2020_FINAL_fbaa038

Flynn_Skibinski_e_appendix_rev_July_2020_FINAL_fbaa038
