# Supplementary material for: Exploring evolution of maximum growth rates in plankton
Source: J Plankton Res. 2020 Sep 4;42(5):497–513. doi: 10.1093/plankt/fbaa038 (PMC7484936; doi:10.1093/plankt/fbaa038)
Supplement: Flynn_Skibinski_e_appendix_rev_July_2020_FINAL_fbaa038 [file flynn_skibinski_e_appendix_rev_july_2020_final_fbaa038.docx]

**Exploring evolution of maximum growth rates in plankton**

Kevin J. Flynn^*1^, David O.F. Skibinski^2^

^1^Plymouth Marine Laboratory, Prospect Place, West Hoe, Plymouth PL1 3DH, UK

^2^Swansea University Medical School, Swansea University, Swansea, SA2 8PP, U.K.

*Correspondence to: KJF@PML.ac.uk

**METHODS APPENDIX**

See Table 1 for the principle variables, as described in the main text, and further information and parameter values in the Supplementary Table. Equations here carry the format Eq.A*x*, while those in the main paper carry the style Eq.*x*.

**Growth of Phytoplankton (Phyto)**

Phytoplankton growth is described as a function of light and abundance of dissolved inorganic nitrogen (N). The model explicitly describes depth-integrated photosynthesis; depending on depth (Dep) and nutrient loading, the phytoplankton biomass self-shades and limits the rate of gross primary production. The photosynthesis-irradiance rate curve (pytq; Eq.A1) references slope alpha_u, the surface irradiance (PFD), and the maximum photosynthetic rate (pqmax) (Flynn 2018). The photosynthetic growth rate of phytoplankton (psq; Eq.A2) is described as a depth integrated photosynthesis rate (based on Smith, 1936) in a water column of a set depth with light attenuation terms att_tot and exatt (= e^(-att_tot)^).

pytq = (alpha_u*PFD*24*60*60)/pqmax Eq.A1

psq = (pqmax*(LN(pytq+SQRT(1+pytq^2))-LN(pytq*exatt+SQRT(1+(pytq*exatt)^2)))/att_tot) Eq.A2

The variable alpha_u is given by the product of the specific slope alpha and the phytoplankton Chl:C ratio (g g^-1^; ChlC). For PFD the factor 24*60*60 converts the photon flux from per second to per day the time unit used in the simulations. The total attenuation (att_tot) is,

att_tot = Dep * [attco_W+(abco_Chl*(ChlC/NC)*Phyto)] Eq.A3

where Dep is optical water depth (mixed layer depth), attco_W is the absorbance coefficient for the growth medium, and NC is the (fixed) mass ratio content of N:C in the phytoplankton biomass.

Variable pqmax is sufficient to enable the phytoplankton growth rate ($\text{µ}_{\text{T}}^{\text{Phyto}}$) to attain the maximum growth rate ($\text{µ}_{\text{mT}}^{\text{Phyto}})$when growing under resource-replete conditions, against the losses due to respiration. Thus,

pqmax = (1 +$\mathrm{CR}_{\mathrm{const}}^{\mathrm{Phyto}}$) *$\text{µ}_{\text{mT}}^{\text{Phyto}}$ * (N/(N + K_g_)) Eq.A4

where $\mathrm{CR}_{\mathrm{const}}^{\mathrm{Phyto}}$ is a constant representing the basal catabolic expense for Phyto. This is indexed to$\text{µ}_{\text{mT}}^{\text{Phyto}}$ under the assumption that a faster growing organism will also have a higher basal rate of respiration in support of catabolic house-keeping.

Nutrient-limitation described in Eq.A4 uses a rectangular hyperbolic term, akin to a Michaelis-Menten function; this makes reference to the nutrient concentration N and the half saturation constant for nutrient-limited growth of Phyto, K_g_. We have shown elsewhere (Flynn *et al*., 2018) that for a given phytoplankton cell allometric and stoichiometric configuration there is a pro rata trade-off between maximum growth rate ($\text{µ}_{\text{mT}}^{\text{Phyto}}$) and the substrate concentration that enables $\text{µ}_{\text{T}}^{\text{Phyto}}$=$\text{µ}_{\text{mT}}^{\text{Phyto}}$/2 (i.e., the nutrient concentration defined by K_g_), where$\text{µ}_{\text{T}}^{\text{Phyto}}$corresponds to GR_pot_ in the general formulation of Eq.2. To incorporate this relationship in a model where the value of $\text{µ}_{\text{mT}}^{\text{Phyto}}$ can change, as a function of $\text{µ}_{\text{m}\text{R}\text{T}}^{\text{Phyto}}$ which is allowed to evolve, we set a reference nutrient affinity index, $\mu_{\mathrm{mKg}}^{\mathrm{Phyto}}$. The value of $\mu_{\mathrm{mKg}}^{\mathrm{Phyto}}$ assumes a default reference maximum growth rate of a division per day (i.e., a growth rate of 0.693 d^-1^) and a default K_g_ = 0.25µM ammonium (=0.25×14 = 3.5 mgN m^-3^). As $\mu_{\mathrm{mKg}}^{\mathrm{Phyto}}$ is considered as constant (with default value 0.198; i.e. 0.693/3.5), this automatically also means that to realise a higher growth rate a higher nutrient concentration must be present to enable $\text{µ}_{\text{T}}^{\text{Phyto}}$=$\text{µ}_{\text{mT}}^{\text{Phyto}}$/2. In the model, the value of K_g_ is thus given by,

K_g_ = $\text{µ}_{\text{mT}}^{\text{Phyto}}$/$\mu_{\mathrm{mKg}}^{\mathrm{Phyto}}$ Eq.A5

The value of the maximum growth rate for the phytoplankton, $\text{µ}_{\text{mT}}^{\text{Phyto}}$, can evolve to higher or lower values than the value set at the beginning of the simulations. A value of 1.4 d^‑1^ would be consistent with the potential for C-fixation set by RuBisCO activity (Flynn and Raven, 2017). However, to facilitate exploration of parameter space the possibility of evolution of higher values of $\text{µ}_{\text{mT}}^{\text{Phyto}}$ is permitted in the simulations shown here.

The gross incremental change in biomass concentration due to growth on each timestep, i.e., dPhyto/dt is then,

Grow^Phyto^ = psq × Phyto Eq.A6

**Gross growth of zooplankton Zoo1 and Zoo2**

Grazers (Zoo1 and Zoo2) acquire their food according to encounter theory, and thus the higher the nutrient loading the more likely the prey abundance is to be high and thus saturating. The zooplankton models are based upon an “eat-to-live” consumer formulation (Flynn, 2018), which centres on the maximum growth rate (here as $\text{µ}_{\text{mT}}^{Zoo1}$ and $\text{µ}_{\text{mT}}^{\text{Zoo2}}$) for zooplankton Zoo1 and Zoo2, respectively) being drivers of the consumer growth description. In this construct, as the consumer becomes stressed and needs more food so the maximum rate of grazing (G_m_) increases.

For Zoo1, the maximum grazing rate is defined as:

$G_{m}^{Zoo1}$ = $\text{µ}_{\text{mT}}^{\text{Zoo1}}\times$(1 +$\mathrm{CR}_{\mathrm{const}}^{Zoo1}$)/(AE*(1 - SDA)) Eq.A7

Here $\mathrm{CR}_{\mathrm{const}}^{Zoo1}$ is a constant representing basal catabolic expense for Zoo1 which, just as for Phyto, is indexed to the maximum growth rate at temperate T (i.e., here to $\text{µ}_{\text{mT}}^{\text{Zoo1}}$). AE is assimilation efficiency and SDA is specific dynamic action. AE is primarily a function of food quality and quantity; in practice AE may decline when food is abundant a relatively high value befits an N-based value (Mitra, 2006). SDA is related to the biochemistry of food digestion, accounting for the proportion of that food type that is respired during anabolic processing; it is considered a constant here in this N-based model.

Grazing is itself described as a linear function of the availabilities of each prey type, limited to a plateau value via a rectangular hyperbolic function of the total biomass ingested (Mitra and Flynn, 2006; Flynn and Mitra, 2016). $\mathrm{Cp}_{\mathrm{Phyto}}^{Zoo1}$and $\mathrm{Cp}_{Zoo1}^{Zoo1}$ as the potential capture rates for predation on Phyto and cannibalisation on Zoo1 are, respectively,

$\mathrm{Cp}_{\mathrm{Phyto}}^{Zoo1}$ = Phyto × $\mathrm{Cri}_{\mathrm{Phyto}}^{Zoo1}$ Eq.A8

$\mathrm{Cp}_{Zoo1}^{Zoo1}$ = Zoo1 × $\mathrm{Cri}_{Zoo1}^{Zoo1}$ Eq.A9

Here $\mathrm{Cri}_{\mathrm{Phyto}}^{Zoo1}$ and $\mathrm{Cri}_{Zoo1}^{Zoo1}$ are capture rate parameters (Mitra and Flynn, 2006) which in reality reflect encounter rates, the optimality of handling that prey type if encountered, the likelihood of prey escape, and also prey palatability (Flynn and Mitra, 2016).

As the sum of capture rates approaches the maximum possible, gut satiation controls ingestion. This control is enacted via Eq.A10, which is a rectangular hyperbolic function making reference to a variable, $K_{I}^{Zoo1}$.

Gpl^Zoo1^ = $G_{m}^{Zoo1}\times$($\mathrm{Cp}_{\mathrm{Phyto}}^{Zoo1}$+$\mathrm{Cp}_{Zoo1}^{Zoo1}$)/($\mathrm{Cp}_{\mathrm{Phyto}}^{Zoo1}$+$\mathrm{Cp}_{Zoo1}^{Zoo1}$+$K_{I}^{Zoo1}$) Eq.A10

The value of $K_{I}^{Zoo1}$, which controls satiation feedback upon grazing, is set by default (Mitra and Flynn, 2006) as,

$K_{I}^{Zoo1}$ = $G_{m}^{Zoo1}$/4 Eq.A11

All else being equal, if $G_{m}^{Zoo1}$ increases so does $K_{I}^{Zoo1}$. The total grazing rate for Zoo1 is then given as:

G^Zoo1^ = MIN [Gpl^Zoo1^, ($\mathrm{Cp}_{\mathrm{Phyto}}^{Zoo1}$+$\mathrm{Cp}_{Zoo1}^{Zoo1}$)] Eq.A12

The net result is that, at high prey abundance, grazing tends to follow the rectangular hyperbola defined by Gpl^Zoo1^, whereas at low prey abundance grazing rate tends to be linear, as defined by ($\mathrm{Cp}_{\mathrm{Phyto}}^{Zoo1}$+$\mathrm{Cp}_{Zoo1}^{Zoo1}$). G^Zoo1^ can be partitioned into grazing rate components attributable to grazing of Zoo1 on Phyto and cannibalisation of Zoo1 on itself,

$G_{\mathrm{Phyto}}^{Zoo1}$= G^Zoo1^×$\mathrm{Cp}_{\mathrm{Phyto}}^{Zoo1}$/($\mathrm{Cp}_{\mathrm{Phyto}}^{Zoo1}$+$\mathrm{Cp}_{Zoo1}^{Zoo1}$) Eq.A13

and

$G_{Zoo1}^{Zoo1}$ = G^Zoo1^×$\mathrm{Cp}_{Zoo1}^{Zoo1}$/($\mathrm{Cp}_{\mathrm{Phyto}}^{Zoo1}$+ $\mathrm{Cp}_{Zoo1}^{Zoo1}$) Eq.A14

The total amounts of grazing from these two sources are then,

$\mathrm{Graz}_{\mathrm{Phyto}}^{Zoo1}$ = $G_{\mathrm{Phyto}}^{Zoo1} \times$ Zoo1 Eq.A15

$\mathrm{Graz}_{Zoo1}^{Zoo1}$= $G_{Zoo1}^{Zoo1}\times$ Zoo1 Eq.A16

Together these define the gross incremental growth in the biomass concentration of Zoo1 at each timestep due to Zoo1 grazing activity.

The formulation for Zoo2 grazing is the same as for Zoo1 but substituting Zoo1 and Zoo2 for Phyto and Zoo1 respectively. The grazing and respiration losses from Zoo1 and Zoo2, are returned as N to support growth of future generations of Phyto. Note that the grazing gains by Zoo1 and Zoo2 are included in the model as losses for the components consumed but these losses are not written here explicitly.

**Losses associated with respiration, assimilation efficiency and specific dynamic action**

For Phyto the rate of loss associated with basal catabolic expense is,

$\mathrm{Loss}_{\mathrm{regT}}^{\mathrm{Phyto}}$= $\mathrm{CR}_{\mathrm{const}}^{\mathrm{Phyto}} \times$ $\text{µ}_{\text{mT}}^{\text{Phyto}}$ Eq.A17

The loss in biomass is thus $\mathrm{Loss}_{\mathrm{regT}}^{\mathrm{Phyto}}$ x Phyto (= $\mathrm{Loss}^{\mathrm{Phyto}}$). As explained above, it is assumed that $\mathrm{CR}_{\mathrm{const}}^{\mathrm{Phyto}}$ is indexed to $\text{µ}_{\text{mT}}^{\text{Phyto}}$ on the argument that a faster growing organism will have a higher catabolic rate of respiration associated with elevated maintenance and enzyme turnover. In one application of the model, where a chemostat is simulated (see below), the effect of enhanced losses at higher values of $\text{µ}_{\text{mT}}^{\text{Phyto}}$ is simulated by raising $\text{µ}_{\text{mT}}^{\text{Phyto}}$ in Eq.A17 to the power of the parameter CR_exp_. This parameter is tested with values of 0.6, 1, and 1.4. In other simulations the parameter CR_exp_ is assigned the value of 1 and thus Eq.A17 applies as written.

For Zoo1 the rate of loss is,

$\mathrm{Loss}_{\mathrm{regT}}^{Zoo1}$ = $\mathrm{CR}_{\mathrm{const}}^{Zoo1}$ × $\text{µ}_{\text{mT}}^{\text{Zoo1}}$ + G^Zoo1^ × ((1-AE)+AE×SDA) Eq.A18

The loss in biomass is thus $\mathrm{Loss}_{\mathrm{regT}}^{Zoo1}$ x Zoo1 (=$\mathrm{Loss}^{Zoo1}$).$\mathrm{CR}_{\mathrm{const}}^{Zoo1}$ is the catabolic respiration constant for Zoo1 and, by analogy with Phyto, higher $\text{µ}_{\text{mT}}^{\text{Zoo1}}$ is associated with higher respiration costs. The formulation for Zoo2 is the same as for Zoo1 but substituting Zoo1 and Zoo2 for Phyto and Zoo1 respectively.

**Further Details on Scope for growth (SfG)**

The definition of scope for growth (Eq.3) can be defined specifically for each of the components, Phyto, Zoo1 and Zoo2:

$\text{SfG}^{\text{Phyto}}$ = (psq - $\mathrm{Loss}_{\mathrm{regT}}^{\mathrm{Phyto}}$) / psq Eq.A19

$\text{SfG}^{Zoo1}$ = (G^Zoo1^ - $\mathrm{Loss}_{\mathrm{regT}}^{Zoo1}$) / G^Zoo1^ Eq.A20

$\text{SfG}^{\text{Zoo2}}$ = (G^Zoo2^ - $\mathrm{Loss}_{\mathrm{regT}}^{Zoo2}$) / G^Zoo2^ Eq.A21

The catabolic stress due to the indexing of CR_const_ to µ_mT_ for all plankton components (Eq.A17 and Eq.A18), results in the evolution of lower µ_mRT_  and SfG: with decreased stress µ_mRT_ and SfG can increase (Fig. 2A).

**Losses due to mortality related to scope for growth (SfG)**

The biomass mortality arising from the rate term M_r_ in Eq.2 is made a downstream function of scope for growth. The SfG related mortality is regarded as a function of the deviation from the maximum value of 1 when SfG is at its highest optimal value. It is derived from expressions relating to deviations from an optimum according to Fisher’s geometric adaptive landscape model (Blanquart *et al*., 2014; Tenaillon, 2014). It is defined as a selection coefficient,

s_SfG_ = 1 – e^(-(mc × (1- SfG))^2^) Eq.A22

Constant mc is a mortality coefficient affecting the magnitude of intrinsic mortality; while being a constant within each simulation, it may differ between components (here as mc_Phyto_, mc_Zoo1_, mc_Zoo2_). The curve described by Eq.A22, gives increasing mortality as SfG declines away from its optimal of 1; its shape can be altered by varying the value of the constant mc, higher values of mc resulting in greater mortality at low SfG.

Coefficient s_SfG_ is redefined as s^Phyto^, s^Zoo1^ and s^Zoo2^ for the three organism components. Thus, after growth over a specified time period the loss in biomass of Phyto which regenerates to N is,

M^Phyto^ = - s^Phyto^ × Phyto - s^Phyto^ × psq × Phyto Eq.A23

Similarly, for Zoo1 and Zoo2 the loss is,

M^Zoo1^ = - s^Zoo1^ × Zoo1 - s^Zoo1^ × G^Zoo1^ × Zoo1 Eq.A24

and

M^Zoo2^ = - s^Zoo2^ × Zoo2 - s^Zoo2^ × G^Zoo2^  × Zoo2 Eq.A25

Note that these losses are not included in the computation of SfG but are regarded as a downstream consequence of SfG.

**Computation of the values of evol^Phyto^**

When the difference between Crit^Phyto^ (↑) and Crit^Phyto^ (↓) is small, as when $\text{µ}_{\text{mRT}}^{\text{Phyto}}$ approaches an equilibrium value, it is to be expected that the selection pressure to change $\text{µ}_{\text{mRT}}^{\text{Phyto}}$ would be weaker and the amount of change as determined by the magnitude of evol^Phyto^ would be smaller. This was investigated as follows in a model which allows evol^Phyto^ to vary in value.

For each of the two prospective scenarios (Eq.4 and Eq.5) a measure related to absolute fitness is calculated as:

$W_{\mathrm{abs}}^{\mathrm{Phyto}}$(↑) = (Phyto + Crit^Phyto^ (↑) )/Phyto Eq.A26

$W_{\mathrm{abs}}^{\mathrm{Phyto}}$(↓) = (Phyto + Crit^Phyto^ (↓))/Phyto Eq.A27

and relative selection coefficients as,

$s_{\mathrm{rel}}^{\mathrm{Phyto}}$(↑) = 1 - $W_{\mathrm{abs}}^{\mathrm{Phyto}}$(↑) /(MAX($W_{\mathrm{abs}}^{\mathrm{Phyto}}$(↑) ,$W_{\mathrm{abs}}^{\mathrm{Phyto}}$ (↓)) Eq.A28

$s_{\mathrm{rel}}^{\mathrm{Phyto}}$ (↓) = 1 - $W_{\mathrm{abs}}^{\mathrm{Phyto}}$(↓) /(MAX($W_{\mathrm{abs}}^{\mathrm{Phyto}}$(↑) ,$W_{\mathrm{abs}}^{\mathrm{Phyto}}$ (↓)) Eq.A29

The maximum of $s_{\mathrm{rel}}^{\mathrm{Phyto}}$(↑) and $s_{\mathrm{rel}}^{\mathrm{Phyto}}$(↓) is taken as the measure of the strength of the difference in selective pressure distinguishing the two scenarios ($s_{\mathrm{rel}}^{\mathrm{Phyto}}$). An equation of a similar form to Eq.31 is then used to compute the value of evol^Phyto^ as follows,

evol^Phyto^ = (-LN(1-$s_{\mathrm{rel}}^{\mathrm{Phyto}}$)/λ^2^) ^0.5^ Eq.A30

which determines that evol^Phyto^ decreases as $s_{\mathrm{rel}}^{\mathrm{Phyto}}$ decreases as function of a constant λ, higher values of λ giving lower evol^Phyto^ at low values of $s_{\mathrm{rel}}^{\mathrm{Phyto}}$. The values of λ used are given in the Supplementary Table. When simulating the dynamics under continuous light, the application of this equation rather than using a fixed value of evol^Phyto^ results in slower approach to the $\text{µ}_{\text{m}\text{R}\text{T}}^{\text{Phyto}}$equilibrium. At equilibrium, the value of evol^Phyto^ attains zero. Although overall the time to the $\text{µ}_{\text{m}\text{R}\text{T}}^{\text{Phyto}}$equilibrium is increased compared with use of a fixed value of evol, the final equilibrium value of $\text{µ}_{\text{m}\text{R}\text{T}}^{\text{Phyto}}$ is not affected. However, with a 12 hour light and 12 hour dark cycle the use of a fixed evol^Phyto^ value usually results in stasis in $\text{µ}_{\text{m}\text{R}\text{T}}^{\text{Phyto}}$ evolution because the increase in $\text{µ}_{\text{m}\text{R}\text{T}}^{\text{Phyto}}$ evolution during daylight is precisely reversed during the night. Thus, by default evol^Phyto^ was allowed to vary as described above applying Eq.A30.

The evolution of $\text{µ}_{\text{mRT}}^{\text{Zoo1}}$ and $\text{µ}_{\text{mRT}}^{\text{Zoo2}}$ was also simulated through the computation and application of a variable evol^Zoo1^ and evol^Zoo2^ using equations analogous to Eq.A26 to Eq.A30.

Higher or lower values of λ might thus be regarded as reflecting lower or higher values of heritability for the character in the context of a quantitative genetics model. A degree of stochasticity could be inserted into the model for future work, for example by selecting evol randomly from a distribution centred about zero, or by allowing occasional changes in evol to simulate sporadic mutation.

**Additional References**

Blanquart, F., Achaz, G., Bataillon, T. *et al*. (2014) Properties of selected mutations and genotypic landscapes under Fisher's geometric model. *Evolution* **68**, 3537-3554. doi:10.1111/evo.12545

Mitra, A. (2006) A multi-nutrient model for the description of stoichiometric modulation of predation in micro- and mesozooplankton. *J. Plankton Res.* **28**, 597-611., doi:10.1093/plankt/fbi144

Smith, E. L. (1936) Photosynthesis in relation to light and carbon dioxide. *Proc. Nat. Acad. Sci. U.S.A.* **22**, 504-511. doi:10.1073/pnas.22.8.504

Tenaillon, O. (2014) The utility of Fisher's geometric model in evolutionary genetics. *Ann. Rev. Ecol. Evol. Sys*. **45**, 179-201. doi:10.1146/annurev-ecolsys-120213-091846
